# *Cinnamomum:* The New Therapeutic Agents for Inhibition of Bacterial and Fungal Biofilm-Associated Infection

**DOI:** 10.3389/fcimb.2022.930624

**Published:** 2022-07-08

**Authors:** Mojtaba Didehdar, Zahra Chegini, Seidamir Pasha Tabaeian, Shabnam Razavi, Aref Shariati

**Affiliations:** ^1^ Department of Medical Parasitology and Mycology, Arak University of Medical Sciences, Arak, Iran; ^2^ Department of Microbiology, School of Medicine, Hamadan University of Medical Sciences, Hamadan, Iran; ^3^ Department of Internal Medicine, School of Medicine, Iran University of Medical Sciences, Tehran, Iran; ^4^ Colorectal Research Center, Iran University of Medical Sciences, Tehran, Iran; ^5^ Microbial Biotechnology Research Center, Iran University of Medical Sciences, Tehran, Iran; ^6^ Department of Microbiology, School of Medicine, Iran University of Medical Sciences, Tehran, Iran; ^7^ Molecular and Medicine Research Center, Khomein University of Medical Sciences, Khomein, Iran

**Keywords:** Cinnamomum, cinnamaldehyde, Candida species, *Pseudomonas aeruginosa*, *Escherichia coli*, *Staphylococcus aureus*, biofilm

## Abstract

Due to the potent antibacterial properties of *Cinnamomum* and its derivatives, particularly cinnamaldehyde, recent studies have used these compounds to inhibit the growth of the most prevalent bacterial and fungal biofilms. By inhibiting flagella protein synthesis and swarming motility, *Cinnamomum* could suppress bacterial attachment, colonization, and biofilm formation in an early stage. Furthermore, by downregulation of Cyclic di‐guanosine monophosphate (c‐di‐GMP), biofilm-related genes, and quorum sensing, this compound suppresses intercellular adherence and accumulation of bacterial cells in biofilm and inhibits important bacterial virulence factors. In addition, *Cinnamomum* could lead to preformed biofilm elimination by enhancing membrane permeability and the disruption of membrane integrity. Moreover, this substance suppresses the *Candida* species adherence to the oral epithelial cells, leading to the cell wall deformities, damage, and leakages of intracellular material that may contribute to the established *Candida’s* biofilm elimination. Therefore, by inhibiting biofilm maturation and destroying the external structure of biofilm, *Cinnamomum* could boost antibiotic treatment success in combination therapy. However, *Cinnamomum* has several disadvantages, such as poor solubility in aqueous solution, instability, and volatility; thus, the use of different drug-delivery systems may resolve these limitations and should be further considered in future investigations. Overall, *Cinnamomum* could be a promising agent for inhibiting microbial biofilm-associated infection and could be used as a catheter and other medical materials surface coatings to suppress biofilm formation. Nonetheless, further *in vitro* toxicology analysis and animal experiments are required to confirm the reported molecular antibiofilm effect of *Cinnamomum* and its derivative components against microbial biofilm.

## Introduction

Multi-Drug Resistant (MDR) microorganisms can pose a serious threat to public health and human life if they cause bacterial infections. As a result, the microorganisms that live in biofilm become increasingly resistant to antibiotics (Jamal et al., 2018). Biofilm can protect its inside cells from the host immune system, antibiotics, and environmental factors; therefore, the biofilm community is easily identifiable in many devices and areas, such as polystyrene, glass, medical devices, bathrooms, and wastewater channels (Donlan and Costerton, 2002; Miquel et al., 2016). In general, the spread of biofilm in the environment and the human body is divided into four stages; 1- an attachment that is managed by different adhesion factors, 2- sessile growth stage that is controlled by different intracellular mediators such as Quorum Sensing (QS) signaling, 3- maturation that modulate through a synthesis of Extracellular Polymeric Substances (EPS) and, finally, 4- detachment (Kostakioti et al., 2013; Saxena et al., 2019).

According to recent studies, various microbial pathogens such as *Staphylococcus aureus*, *Pseudomonas aeruginosa*, *Escherichia coli*, and *Candida* species have the potential ability in biofilm formation and increased antibiotic resistance. Microorganisms with the capability of biofilm formation can escape from the immune system. Antibiotics are incapable of destroying or penetrating the inner layer of the biofilm due to the extracellular matrix’s protection, nutrient limitation, adaptive stress responses, and induction of phenotypic variability (Nuryastuti et al., 2009; Hathroubi et al., 2018; Rizzato et al., 2019). Therefore, due to the alarming occurrence of antibiotic resistance, the unavailability of appropriate antibiotics, and the chronic effects of biofilm-related diseases, new control strategies, and compounds are required that exhibit antimicrobial activity against microbial biofilms (Hong et al., 2015; Saxena et al., 2019).

To this end, alternative solutions to biofilm control, such as the use of nanoparticles (NPs), bacteriophage‐biofilm interactions, QS inhibition, enzymes, and natural products (Plant-derived essential oils), have received further attention. Natural products, including plant extracts, oils, and their derivative compounds, are known to be active against a wide variety of microorganisms and have been used to combat pathogens and infections (Kim et al., 2015; Kargaran et al., 2017; Vasconcelos et al., 2018). *Cinnamomum (Cinnamon)*, a tropical Asian spice and a native plant of Sri Lanka, is extracted from the inner bark of a variety of trees from the *Cinnamomum* genus, including *Cinnamomum camphora*, *Cinnamomum osmophloeum*, *Cinnamomum burmannii*, *Cinnamomum zeylanicum*, *Cinnamomum cassia*, and *Cinnamomum verum* (Vasconcelos et al., 2018).

*Cinnamomum* is one of the common natural products that, in addition to being used in cooking, has received much attention due to its anti-oxidative, cardioprotective, anti-inflammatory, and antimicrobial characteristics in medical applications (Hammer et al., 1999; Yanakiev, 2020). It should be noted that the results of a study published in 2021 showed that cinnamomum at concentrations of 1000-2000 µg/ml has no toxic effects on normal human keratinocyte cell line (Wijesinghe et al., 2021).

Notably, cinnamaldehyde, one of the main *Cinnamomum* ingredients containing about 65% of it, due to its acrolein group (α, β-unsaturated carbonyl moiety), could be related to the antimicrobial activity of *Cinnamomum.* Cinnamaldehyde is not sensitive to common antibiotic resistance despite its strong effect on pathogen infections (Bae et al., 1992). In recent years, in addition to the antimicrobial effect, scientists have been interested in using *Cinnamomum* and its derivative components, especially cinnamaldehyde, to inhibit microbial biofilm (Kosari et al., 2020). In this regard, this review primarily focused on the role of *Cinnamomum* and its derivative compounds in the suppression and elimination of microbial biofilm to facilitate their possible widespread use in clinical practice.

## Inhibitory Effects of *Cinnamomum* on Microorganisms Biofilm

### Pseudomonas aeruginosa

*Pseudomonas aeruginosa* is a significant bacterial pathogen that causes various chronic and acute infections (Bahramian et al., 2019). Recent studies reported a high mortality rate for *P. aeruginosa* infection, especially in patients with underlying conditions such as severe burn injuries, cancer, cystic fibrosis, and nosocomial infections (Mah et al., 2003; Bahramian et al., 2019). Various adhesion factors such as pili, flagella, and biofilms lead to the adhesion and survival of this bacterium on medical devices, water, and diverse surfaces (Remold et al., 2011). Furthermore, *P. aeruginosa* biofilm results in chronic infections due to the increasing resistance to different irradiation treatments, disinfectants, immune systems, and antibiotics (Costerton et al., 1999; Stewart and Costerton, 2001; Mah et al., 2003). In this respect, recent studies reported higher antibiotic resistance in the biofilm community of *P. aeruginosa* compared to the planktonic cells because of antibiotic penetration reduction into the complex polysaccharide matrix (glycocalyx) (Spoering and Lewis, 2001; Ma et al., 2009). Hence, biofilms have increased the prevalence of MDR *P. aeruginosa* strains in recent years, and scientists are looking for new agents to manage it more effectively. After demonstrating appropriate antimicrobial function using various mechanisms, *Cinnamomum* and its derivative compounds have also been considered to destroy microbial biofilms (Vasconcelos et al., 2018).

To this end, *Lakshmanan* et al. reported that cinnamtannin B1, one of the active components of *Cinnamomum tamala*, inhibited biofilm formation and swarming motility of *P. aeruginosa*. Notably, cinnamtannin decreases the expression of *fliC* and *rhlA* associated with the synthesis of flagella protein flagellin and rhamnolipid (Lakshmanan et al., 2019). Swarming is one of the main *P. aeruginosa* virulence factors that aids in surface colonization and infection spread. The association between swarming motility and biofilm formation remains unknown because of conflicting results in the literature (Rampioni et al., 2009; Kerekes et al., 2013).

Moreover, the inhibition of QS was reported as the primary mechanism in inhibiting *P. aeruginosa* biofilm formation by *Cinnamomum*. Four QS systems, including PQS, IQS, Las, and Rhl, are recognized in *P. aeruginosa*. Rhl and Las lead to the main virulence phenotypes and physiological activities and organize nearly 10% of the *P. aeruginosa* genome. Las and Rhl (LasR (Transcription Activator Protein) and RhlR) receptors are stimulated in *P. aeruginosa* by binding to N-oxododecanoyl-L-homoserine lactone and N-butyryl-L-homoserine lactone auto-inducers. Following activation, these receptor proteins form complexes and initiate transcriptional expression further (Mukherjee et al., 2017). According to recent reviews, sub-inhibitory levels of cinnamaldehyde downregulated both the *las* and *rhl* QS systems by repressing the regulatory proteins LasR and RhlR. In addition to decreasing the production of extracellular virulence factors such as pyocyanin, elastase, and protease, this phenomenon suppressed the expression of the rhamnolipid gene and inhibited biofilm formation in *P. aeruginosa* strain PAO1 (PAO1). This study did not detect the exact QS inhibitory function of cinnamaldehyde, but the authors hypothesized that this substance acts as a QS antagonist (Ahmed et al., 2019).

It should be noted that *lasI*, in the *lasI*/*lasR* system, synthesizes 3-oxo-dodecanoyl-homoserine lactone (3-oxo-C12HSL), and this messenger subsequently binds to the cytoplasmic receptor LasR and activates the expression of genes that produce different virulence factors like elastases, proteases, and exotoxin A (Passador et al., 1993). In this regard, a recent investigation reported that whole *Cinnamomum* oil decreased 3-oxo-C_12_HSL levels in the supernatant culture of PAO1 (Kalia et al., 2015). Furthermore, this oil reduced the pyocyanin and alginate production and swarming motility of this bacterium at increasing concentrations (Kalia et al., 2015). Alginate, an essential component of extracellular polysaccharides that code by the *algD* gene, leads to biofilm structure integrity and confers resistance to antimicrobials by preventing entry. Therefore, inhibition of alginate production by *Cinnamomum* oil could repress biofilm maturation (Kalia et al., 2015).

These data support the finding by *Alva* et al., who reported that *C. verum* leaf ethanol extract significantly reduced the expression of the QS-regulatory gene *RhlI*, related to the signal production of N-Butanoyl-L-homoserine lactone (C4-HSL), and other QS-regulated virulence genes like *PiliA, PhzH, FlagA*, *LasB*, and *algD* in a clinical isolate of *P. aeruginosa*. In this regard, the authors detected reduced *P. aeruginosa* ability in producing pyocyanin, elastase, swarming motility, and biofilm formation. Lower concentrations (below 100 mg/L) of *C. verum* compound did not show any toxicity on zebrafish embryos (Alva et al., 2021).

Moreover, the QS-inhibitory effect of cinnamaldehyde in combination with different antibacterial agents was also performed to destroy *P. aeruginosa* biofilm. A recent study reported that cinnamaldehyde repressed the expression of *lasB*, *rhlA*, and *pqsA*; hence, demonstrating a QS-inhibitory effect. The combined use of cinnamaldehyde and tobramycin revealed strong QS inhibitory effects. Furthermore, combination therapy revealed an additive activity of cinnamaldehyde with tobramycin and colistin in the inhibition of PAO1 biofilm and preformed biofilm dispersion compared to the treatment alone (Topa et al., 2020). In another same study, *Kart* et al. reported that the combined use of cinnamaldehyde and ciprofloxacin showed more reduced minimum biofilm eradication concentration than ciprofloxacin alone. In this regard, the authors reported that cinnamaldehyde inhibited QS and alginate production, thereby inhibiting PAO1 biofilm formation and increasing the antibiofilm activity of ciprofloxacin (Kart et al., 2021). As a result of these findings, it is possible that cinnamaldehyde could increase the success of antibiotic treatment in combination therapy by inhibiting QS and thus increasing the susceptibility of bacterial biofilms to an antibiotic; however, this has not been tested.

Additionally, recent examination results also observed the synergism action for *C. tamala* essential oil (CTEO) and commercially available DNase in disrupting young and mature PAO1 biofilms and *P. aeruginosa* clinical isolate. The combined use of DNase and CTEO showed increased efficiency in disrupting the mature biofilms than the CTEO alone. In this respect, although CTEO inhibited QS-associated virulence factor-like alginate production, it demonstrated limited penetration into the biofilms. Hence, when the biofilm scaffold is loosened due to the degradation of extracellular DNA by the action of DNases, it could increase the CTEO penetration to the deeper layer of the bacterial biofilm (Farisa Banu et al., 2017).

**Figure 1:**
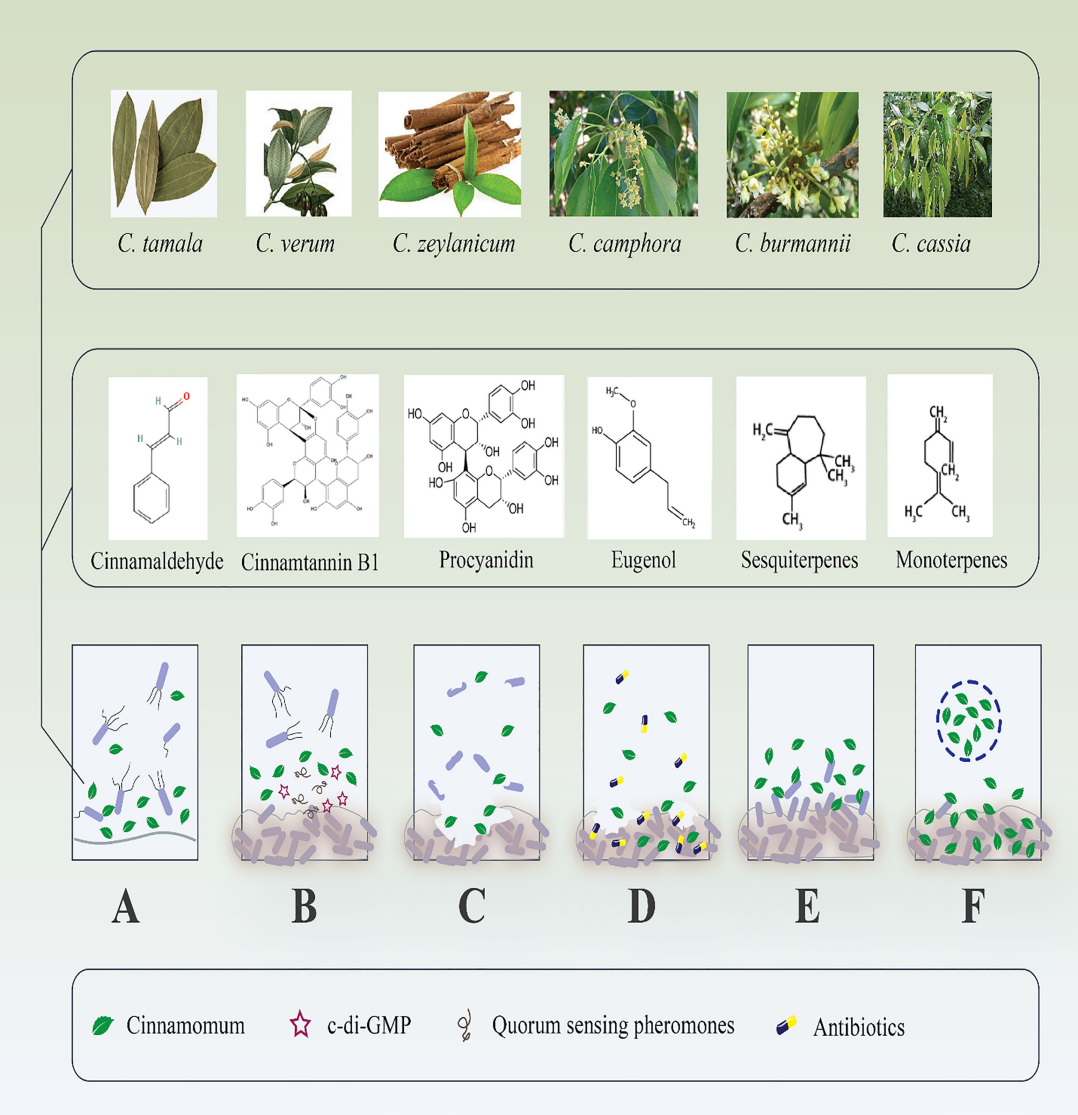
Antibiofilm effects of different species of *Cinnamomum* and their derivate components. **(A)** Inhibition of microbial adhesion to various surfaces. **(B)** Suppression of different bacterial cell signaling mediators that inhibit biofilm maturation. **(C)** Destruction of microbial established biofilm; consequently, **(D)** boost the antibiotic penetration to the dipper layer of the biofilm. **(E)** Handling of recalcitrant infections by repression of new biofilm formation. **(F)** Drug-delivery systems increase the effectiveness of *Cinnamomum* and their derivate components to destroy microbial biofilm.

In addition to the QS-inhibitory effect of *Cinnamomum*, a recent study reported that this compound inhibited Cyclic di‐guanosine monophosphate (c‐di‐GMP) (**Figure 1**) (Topa et al., 2018). C‐di‐GMP is considered a critical cytoplasmic signal and second messenger that controls virulence, cell cycle propagation, motility, and other behaviors, such as biofilm life cycle in several bacteria (Ryan et al., 2006). To this end, *Topa* et al. reported that cinnamaldehyde disrupted transmembrane potential, preformed biofilms, and swarming motility of PAO1. The authors suggested that the cinnamaldehyde carbon atoms may bind to nitrogen-containing components, like protein, in the cytoplasmic membrane, altering the protein structure and losing membrane integrity. Furthermore, the results demonstrated that cinnamaldehyde reduced 66.2% of c‐di‐GMP expression after 5 hours compared to the untreated control (Topa et al., 2018). However, this is the only report of cinnamaldehyde interaction with intracellular c-di-GMP levels; thus, the molecular mechanism by which cinnamaldehyde mediates changes in c-di-GMP levels remains unknown.

Consequently, while the precise mechanism by which *Cinnamomum* acts against the QS system is unknown, it appears to act as a QS and c-di-GMP antagonist. In this regard, *Cinnamomum*, in addition to destroying *P. aeruginosa* biofilm, destroys the bacterium’s virulence factors by inhibiting QS-related factors and c-di-GMP. This phenomenon would allow the host’s innate immunity and other antibiofilm agents to function more successfully. In this respect, synergistic enhancement of antibiofilm agents *via Cinnamomum* administration represents an exciting future development; however, little is known about such effects at the molecular level. As a result, additional research is required to confirm mentioned findings.

### Staphylococcus aureus

In recent years, *S. aureus* with increased antibiotic resistance has increased morbidity, mortality, period of hospitalization, and patient cost. This bacterium results in severe nosocomial infections, and because of the extensive antibiotics usage, it has become the MDR pathogenic bacterium, most threatening to human health (Shariati et al., 2020a). In addition to the frequent occurrence of antimicrobial-resistant strains, *S. aureus* often resides within biofilms at the site of infection (Van den Driessche et al., 2017). Furthermore, *S. aureus* biofilm formation on various medical surfaces, like catheters, is a significant problem in healthcare-associated infections (Ceylan and Ugur, 2015). Accordingly, various antibiotics such as vancomycin and linezolid are used to destroy *S. aureus* biofilm; however, alternatives to the existing antibiotics against methicillin-resistant *S. aureus* (MRSA) biofilm infections are still a subject of interest (Taubes, 2008). In this regard, recent studies in this area have utilized *Cinnamomum* and its derivative compounds.

*García-Salinas* et al. discovered that cinnamaldehyde concentrations greater than 1 mg/mL eliminated the preformed biofilm of *S. aureus* (García-Salinas et al., 2018). In another examination, the *C. zeylanicum* essential oil (CZEO) and its active components, cinnamaldehyde, were used to inhibit *S. aureus* biofilm. Both dramatically decreased biofilm formation on stainless steel and polystyrene surfaces. Hence, the authors suggested that the anti-biofilm efficiency of CZEO is closely linked to cinnamaldehyde (its central component) (Budri et al., 2015). Furthermore, a recent study reported that cinnamaldehyde has a high antibiofilm effect because, after 48 h of treatment, the MRSA biofilms were decreased from approximately 53% to above 82% (Kot et al., 2018). As a result, recent studies have reported *Cinnamomum’s* antibiofilm effects against *S. aureus* and MRSA; however, the exact antibiofilm mechanisms of these substances were not identified in these studies.

In this regard, in other investigations, scientists evaluated molecular antibiofilm mechanisms of *Cinnamomum* and cinnamaldehyde against MRSA. *Kot* et al. reported that cinnamaldehyde efficiently reduced the biofilm formation of MRSA collected from the anus and wounds of hospitalized patients. Moreover, this compound reduced the *fib*, *ebps*, and *eno* genes’ expression levels that encode fibrinogen binding protein, elastin, and laminin-binding protein, respectively. Furthermore, the expression level of polysaccharide intercellular adhesin encoding genes (*icaD*, and *icaA)* decreased after cinnamaldehyde treatment. The authors proposed that by inhibiting *fib*, *ebps*, and *eno*, cinnamaldehyde may have an active role in MRSA adhesion inhibition to fibrinogen present in the blood, elastin, and laminin surfaces. In addition, by reducing the *icaD* and *icaA* expression, cinnamaldehyde could suppress intercellular adherence and accumulation of bacterial cells in biofilm (Kot et al., 2020).

*Jia* et al. also used confocal laser scanning microscopy z-section analyses and reported that cinnamaldehyde, in a dose-dependent manner, disrupted MRSA biofilm and suppressed the expression of *sarA* (Jia et al., 2011). Notably, biofilm-associated protein (Bap) is vital for bacterial adhesion and intercellular accumulation during biofilm formation in icaADBC-independent *S. aureus*. *SarA* regulates the expression of 120 genes in this bacterium and acts as a positive regulator of Bap-mediated biofilm formation. After *bap* gene activation through *sarA*, its expression is closely related to biofilm formation in icaADBC-independent *S. aureus.* Hence, inhibition of *sarA* through cinnamaldehyde could contribute to MRSA biofilm inhibition (Cucarella et al., 2001; Trotonda et al., 2005; Jia et al., 2011).

Finally, CTEO disrupted 60-80% of performed MRSA biofilms in another study. Microscopic examination revealed that CTEO resulted in a reduction in bio-volume and average thickness due to the EPS layer and slime synthesis disruption. Furthermore, this oil reduced MRSA hemolytic activity with a percentage inhibition of 65-80% (Rubini et al., 2018). α–hemolysin, a pore-forming toxin, lyses human red blood cells and also facilitates biofilm formation by regulating cell interactions (Caiazza and O’toole, 2003). According to studies mentioned above, *Cinnamomum*, through downregulation of various MRSA genes, prevents bacterial adhesion to different surfaces and prevents biofilm maturation. However, further *in vitro* and animal experiments are required to confirm the reported molecular interaction of *Cinnamomum* with MRSA biofilm.

The combination of *Cinnamomum* and antibiotics in inhibiting the MRSA biofilm has also demonstrated promising results. A recently published study detected synergistic effects between cinnamaldehyde, β-lactam, and non- β-lactam antibiotics. Cinnamaldehyde suppressed Penicillin-binding proteins (PBP2a) and *mecA*; thus, it is possible that the synergistic effect was caused by the fact that this compound inhibited the *mecA* transcription and translation. Additionally, cinnamaldehyde dramatically reduced the expression of the biofilm regulatory gene *hld*, and subsequently, the MRSA biofilm formation (Wang et al., 2021). Furthermore, *Sundaramoorthy* et al. discovered that their collected *S. aureus* was resistant to all mupirocin concentrations tested. On the other hand, *Cinnamomum* oil significantly eliminated *S. aureus* biofilm. Combining this compound and mupirocin improved the elimination of preformed biofilm compared to the *Cinnamomum* oil applied alone. The authors proposed that this synergistic effect could be associated with the presence of sesquiterpenes and monoterpenes with relative hydrophilicity characteristics in *Cinnamomum* oil that will increase biofilm penetration through the exopolysaccharide matrix. In addition, the hydrophobic nature of phenyl propenes present in this oil may interact with bacterial membrane and penetration (Sundaramoorthy et al., 2021). In this respect, through inhibition of biofilm formation in *S. aureus*, the resistance to antibiotics can be decreased, which may be one of the reasons that antibiotics combined with *Cinnamomum* have a synergistic effect. Therefore, future studies should consider using *Cinnamomum* in combination with antibiotics to destroy MRSA biofilms.

However, *Cinnamomum* essential oil and cinnamaldehyde have several limitations, such as low stability and water solubility. In this regard, in recent years, the use of these substances in various drug delivery systems has been considered (Rai et al., 2017). A recent study encapsulated *Cinnamomum* oil in the liposomes to increase its chemical stability. Afterward, the antibiofilm effect of this conjugation was evaluated against MRSA. The authors reported that liposome encapsulation could release *Cinnamomum* oil slowly, kill MRSA, and destruct its biofilms significantly on various surfaces compared to the essential oil treatment alone. These data suggested that liposome leads to the desired stability and dispersibility of *Cinnamomum* oil and enhances the active time of this compound in the destruction of MRSA biofilms (Cui et al., 2016).

Furthermore, *Meng* et al. used the combination of Gold nanocluster (Au NCs) surface ligand exchange strategy and cinnamaldehyde to inhibit MRSA biofilm. In this regard, cinnamaldehyde was performed on the surface of oxygen species (ROS) generation ability of histidine (His)-stabilized Au NCs. The results indicated that cinnamaldehyde-Au NCs removed significantly more biofilm than Au NCs. In addition, cinnamaldehyde-Au NCs exhibited better antibacterial effects in the pigskin wound infection model. Collected data from the confocal 3D fluorescence microscopy images showed that cinnamaldehyde-Au NCs enhance membrane permeability and lead to membrane integrity disruption and membrane potential dissipation. The antibacterial activity of this combination could be related to the release of the histidine-cinnamaldehyde ligand on the surface of cinnamaldehyde-Au NCs due to the occurrence of a ligand exchange reaction (Meng et al., 2021). As a result, diverse drug-delivery platforms with cinnamaldehyde or *Cinnamomum* could provide novel agents for the destruction of the MRSA biofilm. Finally, it should be noted that other combined uses of *Cinnamomum* and its derivative compounds with various drug-delivery platforms were used to inhibit *S. aureus* biofilm in food industries. These studies have been reported in **Table 1**.

**Table 1:** Previous studies that evaluated the antibiofilm effect of *Cinnamomum* and its active components against different bacterial and fungal biofilm.

Year of publication (References)	*Cinnamomum* species	*Cinnamomum* ingredients	Microorganism	Outcome
2004 **(** Niu and Gilbert, 2004 **)**	NR	Cinnamaldehyde	*Escherichia coli*	Reduced swarming motility and biofilm formation
2008 **(** Brackman et al., 2008 **)**	NR	Cinnamaldehyde	*Vibrio* species	Interfered with auto inducer-2 based QS and inhibited biofilm formation
2009 **(** Nuryastuti et al., 2009 **)**	*C. burmannii* essential oil	NR	*S. epidermidis*	Detached and killed biofilm
2011 **(** Pires et al., 2011 **)**	*C. zeylanicum* essential oil	NR	*Candida parapsilosis*	Inhibited biofilm formation; however, synergistic effect with AMB was not detected.
2011 **(** Amalaradjou and Venkitanarayanan, 2011 **)**	NR	Cinnamaldehyde	*Cronobacter sakazakii*	Inhibited and inactivated biofilms on different surfaces.
2011 **(** Brackman et al., 2011 **)**	*NR*	Cinnamaldehyde	*Pseudomonas aeruginosa Burkholderia cepacia*	Cinnamaldehyde/tobramycin killed bacterial cells in the biofilm.
2012 **(** Khan and Ahmad, 2012 **)**	NR	Cinnamaldehyde	*Candida albicans*	Inhibited the biofilm and showed synergism effect with fluconazole
2012 **(** Nostro et al., 2012 **)**	NR	Cinnamaldehyde	*Listeria monocytogenes, Staphylococcus aureus Escherichia coli* *Staphylococcus epidermidis*	Polyethylene-co-vinylacetate (EVA) films with cinnamaldehyde inhibited biofilm formation.
2013 **(** Upadhyay et al., 2013 **)**	NR	Cinnamaldehyde	*Listeria monocytogenes*	Inhibited biofilm formation on different materials and at various temperatures. Suppressed the expression of the biofilm-associated genes.
2013 **(** Kerekes et al., 2013 **)**	*Cinnamomum* essential oil	NR	*Pseudomonas putida, Escherichia coli*	*Cinnamomum* inhibited the formationmixed culture biofilm.
2014 **(** Sharma et al., 2014 **)**	NR	Cinnamaldehyde	*S. epidermidis*	Cinnamaldehyde in combination with curcumin inhibited biofilm.
2014 **(** Beema Shafreen et al., 2014 **)**	NR	Cinnamaldehyde	*Streptococcus pyogenes*	Showed anti-biofilm effect and decreased *luxS* expression
2014 **(** Piovezan et al., 2014 **)**	*C. zeylanicum* essential oil	Cinnamaldehyde	*Salmonella* Saintpaul	Decreased biofilm activity and viable cells in the mature biofilm.
2014 **(** Zhang et al., 2014 **)**	NR	Cinnamaldehyde	*Staphylococcus aureus and Salmonella* serotype Enteritidis	Suppressed mixed biofilm formation
2015 **(** Liu et al., 2015 **)**	NR	Cinnamaldehyde	*Listeria monocytogenes, Salmonella typhimurium*	Cinnamaldehyde/streptomycin eradicated biofilm.
2015 **(** Duncan et al., 2015 **)**	NR	Cinnamaldehyde	*Escherichia coli* *Pseudomonas aeruginosa, MRSA, and Enterobacter cloacae*	Nanoparticle-stabilized capsules with the cinnamaldehyde that comprises the core of the capsules acts as potent anti-biofilm agents.
2015 **(** Kim et al., 2015 **)**	*Cinnamomum* essential oil	Cinnamaldehyde	*Pseudomonas aeruginosa*	Inhibited swarming motility, hemolytic activity, pyocyanin, and biofilm production.
2016 **(** Karumathil et al., 2016 **)**	NR	Cinnamaldehyde	*Acinetobacter baumannii*	Both substances reduced adhesion and biofilm.
2016 **(** Almeida et al., 2016 **)**	*C. cassia* essential oil	NR	*Candida albicans*	Reduced the accumulation of biofilm.
2016 **(** Singh et al., 2016 **)**	*C. verum*	NR	*Cronobacter sakazakii*	Inhibited the biofilm.
2016 **(** Smith et al., 2016 **)**	NR	Cinnamaldehyde	*Listeria monocytogenes*,	A Bioengineered nisin derivative in combination with cinnamaldehyde eliminated biofilm.
2016 **(** Bassyouni et al., 2016 **)**	*Cinnamomum* essential oil	NR	*Staphylococcus aureus, CoNS, Enterococcus* spp.*, Streptococcus pneumoniae, Moraxella* spp.*, Pseudomonas* spp.*, Klebsiella pneumoniae, Acinetobacter baumannii and Escherichia coli.*	The combination of tobramycin and *Cinnamomum* oil had a synergistic effect on biofilm production.
2016 **(** Hovijitra et al., 2016 **)**	*C. verum or C. zeylanicum essential oils*	NR	*Candida albicans*	Indicated potent fungicidal effect on planktonic and sessile fungus.
2016 **(** Keelara et al., 2016 **)**	NR	Cinnamaldehyde	*Salmonella* species	Decreased biofilm formation.
2017 **(** Kumari et al., 2017 **)**	NR	Cinnamaldehyde	*Cryptococcus species*	Exhibited anti-biofilm activity.
2017 **(** Manukumar and Umesha, 2017 **)**	NR	Cinnamaldehyde	MRSA	Cinnamaldehyde cross-linked low-density polyethylene showed excellent anti-biofilm activity.
2017 **(** Campana et al., 2017 **)**	*C. cassia* essential oil	Cinnamaldehyde	*Staphylococcus aureus*	Oil-based microemulsions disrupted biofilm.
2017 **(** Ramasamy et al., 2017b **)**	NR	Cinnamaldehyde	*Escherichia coli and Pseudomonas aeruginosa Candida albicans* MRSA, MSSA	Cinnamaldehyde immobilized on gold nanoparticles inhibited biofilm formation
2017 **(** Balaure et al., 2017 **)**	*Cinnamomum* essential oil	NR	*Staphylococcus aureus, Candida albicans*	Silica nanoparticles mesoporous nanosystems loaded with *Cinnamomum* essential oil inhibited biofilm.
2017 **(** Ramasamy et al., 2017a **)**	NR	Cinnamaldehyde	*EHEC, MRSA, MSSA* *Pseudomonas aeruginosa*	Cinnamaldehyde loaded to the surface of gold nanoparticles inhibited biofilm.
2017 **(** Lebel et al., 2017 **)**	*C. verum* essential oil	NR	*Solobacterium moorei*	Reduced biofilm formation without cytotoxicity effect on gingival keratinocytes
2017 **(** Rajamanikandan et al., 2017 **)**	NR	Cinnamaldehyde	*Vibrio harveyi*	Cinnamaldehyde could serve as an anti-QS and biofilm formation
2018 **(** Banu et al., 2018 **)**	*C. tamala* essential oil	NR	*Candida* species	Inhibited the biofilm and disrupted EPS.
2018 **(** Aumeeruddy-Elalfi et al., 2018 **)**	*C. zeylanicum* essential oil	NR	*S. epidermidis, E.coli, C. albicans*	A strong anti-biofilm effect was not reported.
2018 **(** Liakos et al., 2018 **)**	*Cinnamomum* essential oil	NR	*Staphylococcus aureus, Pseudomonas aeruginosa, Escherichia coli*, *Candida albicans*	Cellulose acetate - essential oil nanocapsules affected biofilm.
2018 **(** Firmino et al., 2018 **)**	*C. zeylanicum* and *C. cassia* essential oil	Cinnamaldehyde	*Escherichia coli, Pseudomonas aeruginosa*, and *Streptococcus pyogenes*	Both essential oils and cinnamaldehyde showed antibacterial and antibiofilm effects.
2018 **(** Silva et al., 2018 **)**	NR	Cinnamaldehyde	*Salmonella Typhimurium*	Reduced metabolic activity and biofilm biomass.
2018 **(** Vaillancourt et al., 2018 **)**	*C. verum* essential oil	NR	*Staphylococcus hyicus*	Decreased biofilm viability
2019 **(** Maior et al., 2019 **)**	NR	Cinnamaldehyde incorporated into Softone	*Candida albicans*	Inhibited the biofilm
2019 **(** Goc et al., 2019 **)**	*C. cassia* essential oil	Cinnamaldehyde	*Borrelia species*	Eradicate biofilm-like aggregates.
2019 **(** Wang et al., 2019 **)**	*C. camphora* essential oil	Linalool, eucalyptol	*Chomobacterium violaceum*	Decreased violacein and biofilm biomass production.
2019 **(** Wagle et al., 2019 **)**	NR	Cinnamaldehyde	*Campylobacter* *jejuni*	Reduced biofilm formation and inactivated preformed biofilm.
2019 **(** Albano et al., 2019 **)**	NR	Cinnamaldehyde	*Staphylococcus epidermidis*	Suppressed biofilm formation and killed performed biofilm
2019 **(** Balázs et al., 2019 **)**	*Cinnamomum* essential oil	NR	*Haemophilus parainfluenzae*	Pickering nano-emulsion of *Cinnamomum* oil repressed biofilm formation.
2019 **(** Lebel et al., 2019 **)**	*C. verum* essential oil	Cinnamaldehyde	*Streptococcus suis* *Actinobacillus pleuropneumoniae*	A strong anti-biofilm effect was not reported.
2019 **(** Kerekes et al., 2019 **)**	*C. zeylanicum* essential oil	Cinnamaldehyde	*Escherichia coli, Listeria monocytogenes, Pseudomonas putida*, *and Staphylococcus aureus*	Inhibited mono and dual-species biofilm.
2020 **(** Sahal et al., 2020 **)**	*C. verum* essential oil	NR	*Candida tropicalis*	Inhibited the biofilm formation.
2020 **(** Yu et al., 2020 **)**	NR	Cinnamaldehyde	*Campylobacter species*	Inhibited and degraded the biofilm.
2020 **(** Somrani et al., 2020 **)**	NR	Cinnamaldehyde	*Listeria monocytogenes*	Suppressed cell attachment and biofilm formation.
2020 **(** Purkait et al., 2020 **)**	NR	Cinnamaldehyde	*Listeria monocytogenes, Salmonella typhimurium*	Degraded biofilm of both species. cinnamaldehyde/eugenol blend showed the synergistic antibiofilm effect.
2020 **(** Alibi et al., 2020 **)**	*C. verum* essential oil	NR	*Escherichia coli*, *Klebsiella pneumoniae, Acinetobacter baumannii, Pseudomonas aeruginosa, Citrobacter freundii, Klebsiella oxytoca, Salmonella enteridis, Salmonella typhimurium, Salmonella zinzibar, Salmonella livingstone, Salmonella derby, Salmonella heidelberg, Corynebacterium striatum, Staphylococcus aureus*	Indicated anti-biofilm and anti-Qs activities against all isolates.
2020 **(** Condò et al., 2020 **)**	*C. zeylanicum* essential oil	NR	*Escherichia coli*, *Klebsiella pneumoniae, Proteus mirabilis, Pseudomonas aeruginosa*	Destructed mature biofilm.
2020 **(** Sharma et al., 2020 **)**	NR	Cinnamaldehyde	*Staphylococcus* *epidermidis and Escherichia coli*	Cinnamaldehyde/Bacteriocin-GAM217 synergistically increased antibacterial activity against planktonic and biofilm cultures.
2021 **(** Pourkhosravani et al., 2021 **)**	*Cinnamomum* essential oil	NR	*Escherichia coli, Bacillus subtilis*	Reduced adhesion and biofilm.
2021 **(** Liu et al., 2021 **)**	NR	Cinnamaldehyde	*Listeria monocytogenes*	Inhibited biofilm and downregulated Qs-associated genes.
2021 **(** Wijesinghe et al., 2021 **)**	*C. verum* essential oil	Eugenol	*Pseudomonas aeruginosa, Staphylococcus aureus, and Klebsiella pneumoniae*	Decreased biofilm densities without any toxicity on HaCaT cells.
2021 **(** D'agostino et al., 2021 **)**	NR	CIN-102	*Aspergillus* *Fusarium* *Scedosporium*	Inhibited biofilm formation.

NR, not reported; EPS, exopolysaccharide; AMB, amphotericin B; MSSA, methicillin-sensitive Staphylococcus aureus; MRSA, methicillin-resistant Staphylococcus aureus; Qs, Quorum Sensing; CoNS, coagulase-negative Staphylococci; EHEC, Enterohemorrhagic Escherichia coli O157:H7.

### Escherichia coli

*Escherichia coli* is normal flora found in the human and animal digestive tracts (Sack, 2011; Sarowska et al., 2019). Diarrhea is one of the most significant diseases caused by *E. coli*, which leads to the deaths of thousands of people around the world, especially children (Kim et al., 2017). Once this bacterium enters the digestive system, it immediately attaches and colonizes the intestinal cells, evading the host immune system and attacking host cells by producing toxins. In this regard, *E. coli* frequently leads to biofilm-associated opportunistic infections like endometritis, diarrhea, and mastitis (Kim et al., 2017; Wang et al., 2020). Antibiotics can help alleviate disease symptoms and duration, but several *E. coli* species have developed resistance to antibiotics due to antibiotic overuse over the last 50 years (Scotti et al., 2021). Hence, *Cinnamomum* was used to inhibit the attachment and formation of biofilms by *E. coli* to manage infection caused by this bacterium.

*Pourkhosravani* et al. discovered that essential oil extracted from the trunk bark of *Cinnamomum* could inhibit *E. coli* from forming a biofilm. In this regard, anti-adhesion tests performed through crystal violet assay revealed that *Cinnamomum* completely suppressed the adhesion of this bacterium. Furthermore, biofilm metabolic activity and quantification of biofilm biomass showed that *Cinnamomum* suppressed the *E. coli* metabolic activity and biofilm formation by 99% and 100%, respectively. Notably, gas chromatography-mass spectrometry (GC-MS) analysis revealed that E-cinnamaldehyde, α-terpinyl acetate, and copaene accounted for 91.31% of the *Cinnamomum* essential oil (Pourkhosravani et al., 2021). Another investigation also reported that *C. camphora* essential oil (CCEO) killed clinical isolates of *E. coli* from dairy cows with clinical endometritis in both planktonic and biofilm communities. Additionally, the authors evaluated the kinetics of CCEO action against *E. coli* in the suspension and biofilms communities. The results indicated that the bacterial killing occurred most rapidly during the first 5 min of treatment and that the lowest level of viable bacteria was detected nearly 1 h after treatment. These data suggested that the efficiency of CCEO declined over time; thus, the pharmacodynamics time of CCEO was less than 24 h, and repressive effects on the biofilms appeared at an early stage. The microscopic analysis confirmed these results and showed that CCEO firmly suppressed the formation of *E. coli* biofilm, and 4 mL/mL of this essential oil could eliminate the biofilm of this bacterium (Wang et al., 2020).

Recent investigations also corroborated these findings and reported that *Cinnamomum* extract reduced the secretion of EPS and biofilms metabolic activity in a dose-dependent manner, consequently suppressing the *E. coli* strain ATCC 25922 biofilms from 24.45 to 98.09%. On the other hand, the effects on preformed biofilms ranged from 16.20 to 46.14% at various concentrations. The microscopic analysis was consistent with the above findings, indicating that the *Cinnamomum* extract could dramatically hinder and eliminate the *E. coli* biofilms (Lu et al., 2021). Furthermore, *Olszewska* et al. reported that cinnamaldehyde reduced almost 60% of cell metabolic activity and biofilm cell cultivability of *E. coli* strain CECT 434. Notably, the authors suggested that cinnamaldehyde could result in the loss of membrane integrity by biofilm cells by detecting various bacterial cell morphologies such as filamentous cells and weakened coverage of the substratum (Olszewska et al., 2020). A recently published study also reported that *Cinnamomum* extract and cinnamaldehyde inhibited 60% and 86.7% of the biofilm production of *E. coli* isolated from patients with colon cancer, respectively (Kosari et al., 2020). As a result, recent studies have reported *Cinnamomum’s* antibiofilm activity against a variety of *E. coli* isolates; however, the exact antibiofilm mechanism of these substances has not been reported.

Additionally, other researchers evaluated the inhibitory effects of *Cinnamomum* against *E. coli* strain O157:H7 (EHEC) biofilm. This bacterium belongs to the attaching and effacing (A/E) *E. coli* group, leading to bloody diarrhea. Antibiotics should be avoided because they induce the SOS response and activate prophages, resulting in the release of Shiga toxins (Paton and Paton, 1998; Sheng et al., 2016). The EHEC’s ability to adhere to various surfaces and form a biofilm and the absence of effective therapy against EHEC-biofilm-associated infections have led to new antibiofilm agent development. To this end, the results of recent experimentation showed that *C. verum* essential oil (CVEO) inhibited the biofilm formation of EHEC clinical isolates. In addition, the microscopic examination revealed the following characteristics of biofilm cells in the presence of CVEO: sparse microcolonies and individual cells with fewer and shorter interconnecting meshes between cells, but no discernible morphological changes (Scotti et al., 2021).

In another study conducted in 2019, the authors reported that sub-lethal concentrations of cinnamaldehyde increase the expression of *tnaA* and *bssS* genes that are negative regulators of biofilm formation in EHEC (Yuan and Yuk, 2019). Notably, *tnaA* encodes the enzyme tryptophanase that results in indole production and is a signaling molecule that suppresses *E. coli* biofilm formation. Moreover, *BssS* reduces bacterial biofilm formation by affecting cell signaling (Isaacs Jr et al., 1994; Domka et al., 2006). Nevertheless, cinnamaldehyde suppressed the expression of virulence-associated genes, including; Type III secretion systems (T3SSs) (*sepD* and *escC*), flagellar biosynthesis, and functions (*fliA* and *motA*), and chemotaxis (*cheA* and *cheZ*). Afterward, the authors evaluated the association between virulence gene expression changes and observable phenotype alterations. They detected that cinnamaldehyde remarkably decreased the biofilm-forming ability, efflux pump activity, and motility of EHEC, with no induction of antibiotic resistance in the bacterium (Yuan and Yuk, 2019).

Additionally, *Kim* et al. found that *Cinnamomum* bark oil and its constituents inhibited the formation of EHEC biofilms and virulence. Their results demonstrate that coating the biodegradable poly (lactic-co-glycolic acid) surface with cinnamaldehyde or *Cinnamomum* bark oil significantly inhibits EHEC biofilm formation. These compounds inhibited the expression of the *csgAB* and *stx2* genes, which are involved in the formation of curli and the production of Shiga-like toxins, respectively. On the other hand, *Cinnamomum* bark oil did not demonstrate considerable effects on the expression of other biofilm-related genes such as *flhD*, *qseB*, *motB*, and *tnaA* (Kim et al., 2015). Therefore, *Cinnamomum* could inhibit biofilm production of one of the most important *E. coli* pathotypes, EHEC; therefore, it can be used as a preservative in food products. However, additional research is required to determine the precise mechanism by which these substances inhibit biofilm formation.

In addition, *Cinnamomum* demonstrated promising results for inhibiting Uropathogenic *E. coli* (UPEC)-biofilm-associated infections. By creating 80% to 85% of UTI in humans, this bacterium is known as a main etiologic factor for these infections. In addition to various virulence factors, UPEC forms a biofilm that facilitates bacterium growth, toxin secretion, and persistence in excessive pH variation (Flores-Mireles et al., 2015). UPEC biofilms also coat the catheters, in which bacteria embedded in an exopolysaccharide matrix are protected from antimicrobial agents (Manges et al., 2001; Amalaradjou et al., 2010). In this regard, in a previous study, the authors used cinnamaldehyde to treat UPEC biofilm on the polystyrene plates, latex, and urinary catheters. Cinnamaldehyde effectively prevented UPEC biofilm formation on the surfaces mentioned and, when used as an antimicrobial constituent in catheter lock solution, successfully deactivated preformed biofilm. Notably, cinnamaldehyde did not have any cytotoxic effect on bladder epithelial cells (Amalaradjou et al., 2010). This supports the findings by *Kot* et al. that reported cinnamaldehyde at various concentrations hindered the extension of UPEC biofilm on catheter fragments. Additionally, when this substance was used as an antimicrobial constituent in a catheter lock solution, it significantly inactivated preformed UPEC biofilms (Kot et al., 2015).

Another study used Type A procyanidin (TAP) from *C. zeylanicum* to inhibit biofilm formation of MDR UPEC (Vasudevan et al., 2020). Notably, procyanidin is one of *Cinnamomum* components with different biological activities. The results indicated that although TAP treatment did not inhibit the UPEC growth, but affected the biofilm formation. The authors hypothesized that the TAP’s anti-biofilm activity at lower concentrations could be attributed to the pentamer’s four interflavanyl linkages. In addition, TAP downregulated the expression of the *focA*, *papG*, *fimH*, and *fimA*, which mainly manage bacterial adhesion to the urinary tract. Moreover, the synergistic effect between TAP and nitrofurantoin at various pH was detected in this study. Thus, by inhibiting bacterial adhesion, TAP may act as a suppressor of biofilm formation. In addition, the use of this substance may enhance the activity of antibiotics at low concentrations (Vasudevan et al., 2020). As mentioned previously, *Cinnamomum* and its active components could be used to inhibit UPEC because, in addition to the appropriate antibacterial effect, it can also destroy bacterial biofilms. Furthermore, *Cinnamomum* could be a novel antibiofilm agent for catheter surface coatings or an ingredient in catheter lock solutions to prevent catheter-associated UTIs.

### *Candida* species

Oral candidiasis is one of the most prevalent opportunistic infections that lead to oral discomfort, dysgeusia, and pain. Due to the patients’ immunocompromised state, this infection may result in serious complications such as systemic candidiasis and esophageal candidiasis. Most oral candidiasis cases are easily treated with antifungal drugs; however, the conditions could differ in patients with underlying conditions such as HIV and dentures (Williams et al., 2012; Millsop and Fazel, 2016; Swidergall and Filler, 2017). *Candida albicans*, followed by *Candida glabrata*, are the most frequent etiology of oral candidiasis (Miranda-Cadena et al., 2018; Shariati et al., 2020b). The biofilm produced by *C. albicans* is resistant to treatment and outperforms it in the oral cavity. Extracellular DNA and EPS reduce the penetration of antifungals to the biofilm, which is a serious concern that is boosted by the emergence of azole-resistant isolates and the selection of *Candida* species with decreased antifungal susceptibility (Williams et al., 2012). As a result, recent research has focused on *Cinnamomum’s* ability to inhibit *Candida* biofilm formation, thereby limiting the extension of decreasing or resistant antifungal selective pressure.

In a recently published study, *Cinnamomum* oil was used to eliminate mature biofilm of *C. albicans* off dental devices made of heat-polymerized polymethyl methacrylate (PMMA) resin. PMMA is associated with severe candidiasis and oropharyngeal development in patients who wear it. *Cinnamomum* oil destroyed 99% of the *Candida* pre-established biofilm. Furthermore, covering the PMMA samples with this oil for 24 hours also reduced the *C. albicans* biofilm formation by almost 70.0% (Choonharuangdej et al., 2021). Another examination also showed that *C. burmannii* essential oil and its aqueous extract enriched in proanthocyanidins (*Cinnulin*), reduced the fungal adherence to the oral epithelial cells and had an inhibitory effect against preformed *C. albicans* biofilm of clinical isolates. Notably, *Cinnamomum* fractions boosted the oral epithelial barrier integrity and did not show cytotoxicity effects against oral epithelial cells at their effective concentrations. Further, *Cinnulin* decreased the secretion of interleukin (IL)-6 and IL-8 by oral epithelial cells stimulated with TNF-α (Veilleux and Grenier, 2019). Hence, different fractions of *Cinnamomum* could be practical agents for hindering *C. albicans* biofilm and subsequently for managing infections such as *Candida*-infected oral mucositis lesions, oral candidiasis, and denture stomatitis. Additionally, covering dental devices with these substances may be a preventive approach against *Candida* biofilm formation; however, more specific studies are required.

The findings of a recently published study also demonstrated severe antifungal function for cinnamaldehyde against *Candida* species isolated from patients with oral candidiasis. Further, cinnamaldehyde lowered the biomass and metabolic activity of mature biofilm (Miranda-Cadena et al., 2021). Collectively, the biofilm biomass reduction could play a key role in controlling MDR infections as biofilms are a source for dispersal of cells with beneficial features such as forming new biofilms and enhancing virulence plus adhesion (Uppuluri et al., 2010; Nobile and Johnson, 2015).

In addition to cinnamaldehyde, eugenol was also reported as a central component of *Cinnamomum* for inhibiting *Candida* biofilm. Eugenol is a phenylpropanoid detected in aromatic plants, especially as the main ingredient in clove oil (Marchese et al., 2017). In a 2020 study, researchers reported that CVEO indicated remarkable antifungal potency against 24-h preformed *Candida* species biofilms. Exposure to the CVEO could lead to cell wall deformities as well as leakages of intracellular materials in *Candida* biofilm. None of the CVEO-tested concentrations in this study showed any cytotoxicity on human non-cancer keratinocytes. GC-MS evaluation illustrated eugenol as the main component of CVEO (Wijesinghe et al., 2021).

*Wijesinghe* et al. also reported eugenol as the main compound (77.22%) of CVEO. This essential oil significantly suppressed germ tube formation, adhesion, and biofilm formation in common *Candida* species strains. Microscopic analysis also revealed CVEO treatment lead to leakage of intracellular materials as well as cell wall damages and deformities, plus cell density reduction for biofilm cells. The *Galleria mellonella* larvae experiment model did not exhibit any cytotoxicity for CVEO (Wijesinghe et al., 2020). Finally, another investigation revealed that CZEO suppressed biofilm formation and considerably decreased *Candida* monospecies along with multi-species preformed biofilm at 24 h and 48 h, respectively. Chemical assessment identified eugenol as the primary component (68.96%) of CZEO and confirmed previous findings. In addition, this essential oil showed low cytotoxicity effects against peripheral mononuclear and red blood cells (Rangel et al., 2018).

Eugenol could have a promising role in the degradation of *Candida* biofilms. Nevertheless, eugenol’s precise antifungal and antibiofilm activity has not been determined in the mentioned studies, and additional molecular and *in vitro* investigations are needed. Collectively, *Cinnamomum* species and plant material used for extraction could produce oils with different major components, suggesting that the anti-biofilm effect of each component should be evaluated separately.

On the other hand, some studies have evaluated the molecular interactions of *Candida* cells in biofilm community with *Cinnamomum* and cinnamaldehyde and discovered interesting results. A study performed by *El-Baz* et al. reported that CVEO has an inhibitory effect against *C. albicans* biofilm isolated from different clinical samples. This essential oil also suppressed the hemolysin and phospholipase activity of this fungus. Microscopic images also described the diminished biofilm formation in terms of suppressed adhesion. Note that according to molecular docking, cinnamaldehyde, as the main component of CVEO, has an impact on Als3 (El-Baz et al., 2021). The Als adhesive proteins are one of the most extensively studied virulence characteristics of *C. albicans*, where deletion of Als3 led to a remarkable decrease in fungal adhesion (Hoyer and Cota, 2016). Hence, the Als3 interaction and cinnamaldehyde may be a promising result for using these compounds to inhibit *C. albicans* adhesion and biofilm formation (El-Baz et al., 2021).

In this regard, another study discovered that cinnamaldehyde destroyed *Candida* cellular development and suppressed biofilm formation by detecting specific features such as the expression of rare pseudo-hyphae and absence of chlamydoconidia. Molecular docking evaluation indicated negative ligand-receptor interaction for cinnamaldehyde with the most affinity for squalene thymidylate synthase and epoxidase. Thus, the authors hypothesized that cinnamaldehyde could restrict the formation of biofilms in *Candida* by affecting important targets present in the fungal cell and nucleus; however, further docking studies are required for precise identification (Da Nóbrega Alves et al., 2020).

Furthermore, *Gupta* et al. discovered that cinnamaldehyde could destroy the biofilm community of *C. glabrata* clinical isolates from biomaterials’ surfaces such as contact eye lens and urinary catheter. Furthermore, cinnamaldehyde could increase ROS production, cell lysis, and plasma membrane ergosterol content. However, this compound suppressed *C. glabrata* enzymes’ activity such as phospholipase, catalase, and proteinase. Detailed molecular analysis showed that cinnamaldehyde downregulated the expression of *FKS1*, *AUS1*, *KRE1*, and *CDR1* genes related to the 1,3-β-glucan synthase sterol importer, GPI-anchored protein, and multi-drug transporter, respectively. In this regard, the authors proposed that ergosterol interaction with cinnamaldehyde would change the integrity and permeability of the cell membrane, and ultimately result in intracellular content leakage and cell lysis (Gupta et al., 2018). Thus, the interaction of cinnamaldehyde with different *Candida* cellular pathways could suppress various virulence phenotype of this fungus like biofilm community. Accordingly, the data on this subject are scarce, necessitating additional research.

## Biofilm-Associated Dental Disorders

### Dental Surface Biofilm

Some of the most prevalent dental disorders like periodontitis, endodontic failure, and dental caries contribute to the biofilm formation of different bacteria (Sun et al., 2013; Jhajharia et al., 2015). Dental caries is known as the most significant chronic and costly oral disorder affecting the health of children and adults worldwide (Ren et al., 2016). *Streptococcus mutans* are customarily found in various oral cavity sites and are the most common bacterium related to the initiation of dental caries (Lynch et al., 2013; Alshahrani and Gregory, 2020). By fermenting dietary carbohydrates, this bacterium, mainly sucrose, leads to the production of extracellular polysaccharides with high adhesion ability to the tooth surface. As a result, *S. mutans* may play a role in dental caries by producing a biofilm on the tooth surface f (Koo et al., 2010; Cheon et al., 2013; Klein et al., 2015). Various approaches, such as mechanical cleaning and chemical plaque control, are performed to destroy bacterial biofilm from the dental surface. However, certain limitations, such as an unpleasant taste, staining on the teeth, and the development of antimicrobial-resistant strains due to long-term use of specific antimicrobial agents, have prompted scientists to seek suitable alternative methods (Wilson and Patterson, 2008; Malhotra et al., 2011; Wiwattanarattanabut et al., 2017). Accordingly, recent studies used *Cinnamomum* and cinnamaldehyde to destroy *S. mutans* biofilm.

*Alshahrani* et al. reported that the water extract from *C. burmannii* could suppress *Streptococcus mutans* biofilm formation (Alshahrani and Gregory, 2020). Another investigation reported that CZEO inhibited the biofilm formation of *S. mutans* by up to 80% and reduced 50% of the 24-hour pre-established biofilm of this bacterium (Wiwattanarattanabut et al., 2017).

Molecular interaction of cinnamaldehyde and *S. mutans* biofilm have been reported in two recently published studies. The results of one of them demonstrated that cinnamaldehyde reduced *S. mutans* biofilm metabolism and biomass. Notably, cinnamaldehyde enhances hydrophobicity and reduces *S. mutans* aggregation, reducing acid production and acid tolerance. Hence, it is possible that cinnamaldehyde could suppress bacterial adherence to the tooth surfaces, and consequently, inhibit biofilm formation. Furthermore, the authors suggested that cinnamaldehyde, through inhibition of glycolytic enzymes present in the acid production pathway, may impair bacterial acidogenicity and reduce tooth demineralization. Finally, cinnamaldehyde downregulated the expression of various biofilm-associated genes such as *vicR*, *ciaH*, *ciaH*, and *gtf* cluster genes (He et al., 2019). This supports the findings of the *Balasubramanian* et al. study, which found that cinnamaldehyde significantly inhibited acid production and biofilm formation by *S. mutans*. Furthermore, the results revealed that cinnamaldehyde impaired the expression of genes related to bacteriocins production, QS, stress tolerance, metabolism, and biofilm formation in *S. mutans*. As a result, these data recommend that cinnamaldehyde, in addition to biofilm destruction, could suppress the various virulence factors of *S. mutans*. (Balasubramanian et al., 2021).

In this respect, the appropriate concentration of *Cinnamomum* and cinnamaldehyde in oral hygiene products such as dental floss, mouthwashes, and toothpaste could lead to the repressive of bacterial biofilm and caries incidence reduction. However, more investigations are required to understand better the molecular mechanism underlying the inhibitory effect of cinnamaldehyde on *S. mutans* biofilm formation. Furthermore, the inhibitory effect of *Cinnamomum* and cinnamaldehyde should be evaluated against multi-species dental surface biofilm. Because diverse species with varying antibiotic resistance patterns coexist in this type of biofilm, biofilm formation increases their tolerance to antibacterial agents (Worreth et al., 2021). Accordingly, a direct comparison of *Cinnamomums’* inhibitory effect on mono and multi-species biofilms is not possible, and future research should place a greater emphasis on multi-species biofilms. It is worth noting that the other experiments in which *Cinnamomum* and its derivatives were used to inhibit dental surface biofilm formation are listed in **Table 2**.

**Table 2:** Antibiofilm effect of *Cinnamomum* and its derivative compounds against biofilm of bacteria associated with dental disorders.

Year of publication (references)	*Cinnamomum* species	*Cinnamomum* ingredients	Microorganism	Biofilm model	Outcome
2021 **(** Worreth et al., 2021 **)**	NR	Cinnamaldehyde	*Streptococcus mutans Streptococcus mitis*	Cellulose-based material	Decreased bacterial growth and biofilm formation on cellulose-based dental clear aligners.
2021 **(** Mala et al., 2021 **)**	*Cinnamomum*	NR	*Streptococcus mutans*	Microplates	Inhibited biofilm formation
2021 **(** Dos Santos et al., 2021 **)**	NR	Cinnamaldehyde	*Streptococcus mutans Lactobacillus casei Fusobacterium nucleatum* *Actinomyces israelii* *Enterococcus faecalis*	Microplates	Curcumin- cinnamaldehyde hybrids showed an antibiofilm effect against oral pathogens.
2021 **(** Jeong et al., 2021 **)**	*C. verum* EO nanoemulsion	NR	Aciduric bacteria that cause dental caries.	Microcosm biofilm model	Suppressed oral microorganisms’ growth in biofilms and multi-species oral biofilms maturation.
2020 **(** De Oliveira Carvalho et al., 2020 **)**	*C. zeylanicum* EO	NR	*Streptococcus mutans*	Microplates	The *C. zeylanicum* EO antibiofilm activity against *S. mutans* was not significant compared to the control.
2018 **(** Ribeiro et al., 2018 **)**	NR	Citronellol, cinnamic acid, and cinnamaldehyde	*Streptococcus mutans*	Microplates	These substances inhibited the planktonic and biofilm community of *S. mutans*
2018 **(** Wang et al., 2018 **)**	*C. zeylanicum* EO	Cinnamaldehyde	*Porphyromonas gingivalis*	Microplates	*C. zeylanicum* EO and cinnamaldehyde suppressed biofilm formation of *P. gingivalis* by 74.5% and 67.3%, separately. However, only *C. zeylanicum* EO reduced preformed biofilms by 33.5%.
2017 **(** Zaltsman et al., 2017 **)**	NR	Cinnamaldehyde-modified particles	*Streptococcus mutans*	Resin material	This particle showed antibiofilm activity.
2005 **(** Filoche et al., 2005 **)**	*Cinnamomum*	NR	*Streptococcus mutans, Lactobacillus plantarum*	Microplates	The chlorhexidine amount needed to inhibit the bacterial biofilm was decreased in combination with *Cinnamomum.*

NR, not reported; EO, essential oil.

### Root Canal Biofilm

Bacterial removal from the root canal system is the most critical aspect of root canal treatment success (Sjögren et al., 1997). *Enterococcus faecalis* has a potential role in root canal treatment failure. This may be due to the significant *E. faecalis* potency to resist and attach to treated dentine surfaces and its ability to tolerate nutrient-deprived environments encountered inside root canals. Moreover, in addition to high antibiotic resistance characteristics, this bacterium can form biofilms on various substrates, such as hydroxyapatite, gutta-percha, dentin, and bone (Liu et al., 2010; Xu et al., 2019; Marcoux et al., 2020). In this regard, *E. faecalis* contributes to various peri-radicular lesions, including primary and secondary endodontic infections (Evans et al., 2002). Diverse antibacterial strategies like intracanal medicaments, diverse instrumentation techniques, and the systemic and local application of antibiotics have been used to control persistent infections. Nevertheless, these common methods are not always effective, and the systemic administration of antibiotics could exhibit several adverse effects such as allergic reactions, toxicity, and development of bacteria with higher antibiotic resistance features (Hoelscher et al., 2006; Mohammadi and Abbott, 2009; Saber and El-Hady, 2012).

Additionally, previous research has demonstrated that conventional disinfectants such as chlorhexidine and hypochlorite are incapable of completely eradicating the microbial community and bacterial biofilm from the root canal (Neelakantan et al., 2017; Ali et al., 2020). Therefore, although root canal infections are polymicrobial, *E. faecalis* is the most prevalent bacterium isolated in failed treatments and is thus considered the model organism to evaluate the effect of new agents [14]. In this regard, recent studies used *Cinnamomum* and cinnamaldehyde to eliminate *E. faecalis* biofilm.

*Gupta* et al. used an extract of *C. zeylanicum* to inhibit the growth of an *E. faecalis* biofilm. When applied to biofilms formed on cellulose nitrate membranes and tooth substrates, this substance kills all bacteria. However, the extract of *C. zeylanicum* used in this study was less effective against *E. faecalis* than sodium hypochlorite (NaOCl) (Gupta et al., 2013). Another investigation reported that intracanal application of CZEO for 14 days completely eliminated *E. faecalis* biofilm and was non-cytotoxic for L929 fibroblasts. Notably, GC-MS analysis showed that cinnamaldehyde was the main component of CZEO (Abbaszadegan et al., 2016).

Additionally, *Abbaszadegan* et al. found that CVEO killed 90.4% of *E. faecalis* cells embedded in biofilms, compared to 31.1% for chlorhexidine. The authors suggested that CVEO’s high efficiency could be attributed to its high terpene content, which is known for its high hydrophobicity and volatility, as well as its low molecular weight (Marcoux et al., 2020). Furthermore, the results of a 2020 study demonstrated that cinnamaldehyde significantly reduced biofilm formation and prevented biofilm recovery in a clinical strain of *E. faecalis* isolated from failed root canal treatment. Cinnamaldehyde treatment for 15 min had the same effect on biofilm metabolic activity as 2% chlorhexidine and 1% sodium hypochlorite. Besides, 24 h treatment with cinnamaldehyde was significantly more effective than 2% chlorhexidine at reducing biofilm viable cell counts. Notably, cinnamaldehyde inhibited the *E. faecalis* biofilms recovery as there was no significant enhancement in the bacterial count at day ten compared to day 0 (Ali et al., 2020). The authors suggested that the antibiofilm capacity of cinnamaldehyde could be related to its penetration and destruction of the *E. faecalis* hydrophobic cell membrane. Consequently, cell membrane injuries lead to intracellular contents’ leakage and suppression of the membrane-bound ATPase activity (Vasconcelos et al., 2018; Ali et al., 2020).

Finally, recently published work interestingly reported that cinnamaldehyde, at sub-inhibitory concentration, suppressed the production of exopolysaccharides and biofilm formation of *E. faecalis* and reduced its hemolytic and proteolytic activity. On the other hand, the authors did not observe this prohibitory effect for cinnamaldehyde against biofilm of two strains of *E. faecalis* with *fsrB* and *fsrC* genes insertion-deletion. Furthermore, cinnamaldehyde considerably downregulated *fsrB* and *fsrC* expression (Ali et al., 2021). It should be noted that recent studies indicated that the Fsr QS system by production of gelatinase related to the virulence and biofilm formation of *E. faecalis*. In this regard, the *fsrB* gene encodes a transmembrane protein that processes a propeptide to generate a peptide pheromone. In addition, *fsrC* encodes a histidine kinase sensor that responds to the peptide-signaling molecule, phosphorylates its response regulator, and subsequently induces the *gelE-sprE* operon’s transcription (Nakayama et al., 2006). Thus, these data suggested that cinnamaldehyde inhibits the formation of *E. faecalis* biofilms by targeting the Fsr QS system; however, additional complimentary research is required to confirm this hypothesis.

As a result, *Cinnamomum* and cinnamaldehyde may inhibit the formation of *E. faecalis* biofilms; thus, they may be used in endodontics to control root canal flora. However, possible interactions of these substances with the physical, chemical and pharmacological characteristics of root canal filling materials are still obscure. In addition to *E. faecalis*, other microorganisms such as *Fusobacterium nucleatum*, *Porphyromonas endodontalis*, *Prevotella intermedia*, *Actinomyces israelii*, and *C. albicans* are present in the root canal that may have a potential effect on *Cinnamomums’* repressive effect. Therefore, additional research should be conducted on *Cinnamomum’s* anti-biofilm effect on multi-species biofilms in various environmental conditions and clinically relevant models, such as the whole tooth model for the biofilm assay.

## Conclusion

*Cinnamomum* and its derivatives, particularly cinnamaldehyde, have demonstrated promising anti-biofilm properties against various microorganisms. As a result, it may be used in place of antibiotics to treat biofilm-related infections. Although some studies have demonstrated that *Cinnamomum* has molecular interactions with the cellular pathways of microorganisms, additional research is required to substantiate these findings. Additionally, animal models, clinical trials, and a precise assessment of cell cytotoxicity caused by long-term exposure to *Cinnamomum* are required.

## Author Contributions

AS and MD conceived and designed the study. AS and ZC contributed to comprehensive research. ZC, AS, and MD wrote the paper. SR and ST participated in manuscript editing. All authors contributed to the article and approved the submitted version.

## Conflict of Interest

The authors declare that the research was conducted in the absence of any commercial or financial relationships that could be construed as a potential conflict of interest.

## Publisher’s Note

All claims expressed in this article are solely those of the authors and do not necessarily represent those of their affiliated organizations, or those of the publisher, the editors and the reviewers. Any product that may be evaluated in this article, or claim that may be made by its manufacturer, is not guaranteed or endorsed by the publisher.

## Abbreviations

c‐di‐GMP, Cyclic di‐guanosine monophosphate; MDR, Multi-Drug Resistant; QS, Quorum Sensing; EPS, Extracellular Polymeric Substances; NPs, nanoparticles; 3-oxo-C12HSL, 3-oxo-dodecanoyl-homoserine lactone; CTEO*, C. tamala* essential oil; MRSA, methicillin-resistant *S. aureus*; CZEO*, C. zeylanicum* essential oil; Bap, biofilm-associated protein; PBP2a, Penicillin-binding proteins; GC-MS, gas chromatography-mass spectrometry; CCEO*, C. camphora* essential oil; CVEO*, C. verum* essential oil; TAP, Type A procyanidin; UPEC, Uropathogenic *E. coli*; PMMA, Polymerized Polymethyl Methacrylate.
